# Young adult cancer risk behaviours originate in adolescence: a longitudinal analysis using ALSPAC, a UK birth cohort study

**DOI:** 10.1186/s12885-021-08098-8

**Published:** 2021-04-07

**Authors:** Caroline Wright, Jon Heron, Ruth Kipping, Matthew Hickman, Rona Campbell, Richard M. Martin

**Affiliations:** 1 Department of Population Health Sciences, Population Health Sciences, Bristol Medical School, University of Bristol, BF4, Barley House, Oakfield Grove, Bristol, BS8 2BN UK; 2 MRC Integrative Epidemiology Unit (IEU), Population Health Sciences, Bristol Medical School, University of Bristol, Bristol, UK; 3 National Institute for Health Research (NIHR) Bristol Biomedical Research Centre, University Hospitals Bristol NHS Foundation Trust and the University of Bristol, Bristol, UK

**Keywords:** Cancer risk behaviours, Adolescence, ALSPAC, UK birth cohort study, Early adulthood, Longitudinal latent class analysis

## Abstract

**Background:**

An estimated 40% of cancer cases in the UK in 2015 were attributable to cancer risk behaviours. Tobacco smoking, alcohol consumption, obesity, and unprotected sexual intercourse are known causes of cancer and there is strong evidence that physical inactivity is associated with cancer. These cancer risk behaviours co-occur however little is known about how they pattern longitudinally across adolescence and early adulthood. Using data from ALSPAC, a prospective population-based UK birth cohort study, we explored patterns of adolescent cancer risk behaviours and their associations with cancer risk behaviours in early adulthood.

**Methods:**

Six thousand three hundred fifty-one people (46.0% of ALSPAC participants) provided data on all cancer risk behaviours at one time during adolescence, 1951 provided data on all cancer risk behaviours at all time points. Our exposure measure was quartiles of a continuous score summarising cumulative exposure to cancer risk behaviours and longitudinal latent classes summarising distinct categories of adolescents exhibiting similar patterns of behaviours, between age 11 and 18 years. Using both exposure measures, odds of harmful drinking (Alcohol Use Disorders Identification Test-C ≥ 8),daily tobacco smoking, nicotine dependence (Fagerström test ≥4), obesity (BMI ≥30), high waist circumference (females: ≥80 cm and males: ≥94 cm, and high waist-hip ratio (females: ≥0.85 and males: ≥1.00) at age 24 were estimated using logistic regression analysis.

**Results:**

We found distinct groups of adolescents characterised by consistently high and consistently low engagement in cancer risk behaviours. After adjustment, adolescents in the top quartile had greater odds of all outcomes in early adulthood: nicotine dependency (odds ratio, OR = 5.37, 95% confidence interval, CI = 3.64–7.93); daily smoking (OR = 5.10, 95% CI =3.19–8.17); obesity (OR = 4.84, 95% CI = 3.33–7.03); high waist circumference (OR = 2.48, 95% CI = 1.94–3.16); harmful drinking (OR = 2.04, 95% CI = 1.57–2.65); and high waist-hip ratio (OR = 1.88, 95% CI = 1.30–2.71), compared to the bottom quartile. In latent class analysis, adolescents characterised by consistently high-risk behaviours throughout adolescence were at higher risk of all cancer risk behaviours at age 24, except harmful drinking.

**Conclusions:**

Exposure to adolescent cancer risk behaviours greatly increased the odds of cancer risk behaviours in early adulthood. Interventions to reduce these behaviours should target multiple rather than single risk behaviours and should focus on adolescence.

**Supplementary Information:**

The online version contains supplementary material available at 10.1186/s12885-021-08098-8.

## Background

Cancer is one of the leading causes of death in the UK [1] and has superseded cardiovascular disease as the leading cause of death in high income countries [2]. Tobacco smoking, [3, 4] alcohol consumption, [3, 5–8] obesity, [3, 5, 9–11] and unprotected sexual intercourse [3, 12] are known causes of cancer. There is also strong evidence that physical inactivity is associated with cancer incidence, [5, 13] with emerging findings indicating a causal association between physical inactivity and prostate, colorectal and breast cancers [14–16]. These exposures are also known predictors of other adverse health outcomes and a significant burden to the NHS. In 2017–18, there were an estimated 489,300 hospital admissions and 77,800 deaths attributable to tobacco smoking, representing 16% of all deaths in the UK [17] and 10,660 hospital admissions with a primary diagnosis of obesity [18]. In 2018/19 there were 358,000 estimated admissions to hospital and 5698 alcohol-specific deaths, where the main reason for admission was attributable to alcohol [19]. While obesity is not a behaviour, for ease of description, we will refer to this group of exposures as behaviours henceforth.

Experimentation constitutes a normal part of growing up. However, these behaviours’ can become habitual and set the pattern for a life characterised by unhealthy practices [20]. While there have been significant declines in adolescent tobacco smoking and alcohol consumption in the UK, [21] the prevalence of other risk behaviours remains high. Twenty-eight per cent of children aged 2 to 15 years are overweight and among them, 17% of boys and 15% of girls are obese with prevalence increasing with age [22]. Physical inactivity and sedentary behaviour are common with only 18% of 5–16 years olds in England meeting current Chief Medical Officer guidelines of taking part in sport and physical activity for at least 60 min every day [18]. Young people also have the highest diagnosed rates of the most common sexually transmitted infections (STIs) of all age groups [23].

There is evidence that these behaviours co-occur in cross-sectional data, [24–27] there is also evidence that single risk behaviours co-occur in longitudinal data, [28–33] however less is known about how this multiplicity of risk behaviours pattern longitudinally across adolescence and into early adulthood. The aim of this study was to investigate the patterns of multiple cancer risk behaviours across adolescence (age 11–18 years) and their associations with subsequent cancer risk behaviours in early adulthood (age 24 years). Our primary hypothesis was that adolescents engaged in more cancer risk behaviours across adolescence would be at greater risk of early adult cancer risk behaviours at age 24 years. We have previously shown in cross-sectional analyses that multiple risk behaviours cluster by number of behaviours rather than producing distinct risk profiles [27]. Therefore, our secondary aim was to explore, in a longitudinal analysis, if multiple cancer risk behaviours cluster across adolescence to produce qualitatively distinct risk profiles, characterised by engagement in certain behaviours. To our knowledge this is the first study to develop longitudinal measures of multiple adolescent behaviours and explore associations with cancer risk outcomes in early adulthood.

## Methods

### Aims of the study

Using longitudinal data from the Avon Longitudinal Study of Parents and Children (ALSPAC), an ongoing prospective observational population-based birth cohort study the aims of this study were: (i) to investigate the patterns of multiple cancer risk behaviours across adolescence (age 11–18 years) using both quartiles of a continuous score summarising cumulative exposure and longitudinal latent class analysis; and (ii) to explore whether and how these patterns are associated with subsequent cancer risk behaviours in early adulthood (age 24 years).

### Design & setting of the study

Data were drawn from ALSPAC, an ongoing prospective observational population-based birth cohort study investigating the effects of a wide range of influences on health and development across the life course [34, 35]. Pregnant women, resident in Avon, UK and with expected dates of delivery 1st April 1991 to 31st December 1992 were invited to take part in the study. The initial number of pregnancies enrolled was 14,541 (for these at least one questionnaire has been returned or a “Children in Focus” clinic had been attended by 19/07/99). Of these initial pregnancies, there was a total of 14,676 foetuses, resulting in 14,062 live births and 13,988 children who were alive at 1 year of age. Details of all available questionnaires and data can be found through a searchable data dictionary (http://www.bristol.ac.uk/alspac/researchers/our-data/). Ethical approval for the study was obtained from the ALSPAC Law and Ethics Committee and local Research Ethics Committees. Informed consent for the use of data collected via questionnaires and clinics was obtained from participants following the recommendations of the ALSPAC Ethics and Law Committee at the time.

### Exposure measure - adolescent cancer risk behaviours

We used repeated measures of tobacco smoking, alcohol consumption, obesity, sexual risk and physical inactivity at ages ~ 11, ~ 14, ~ 16 and ~ 18 years (see Table 1). We include both physical inactivity and obesity as exposures because although physical activity may lie on the causal pathway to obesity, [36] there is strong evidence that both exposures have their own unconfounded, causal effect on cancer outcomes [14–16, 37]. Self-completed questionnaires issued during clinics, self-completed responses to postal questionnaires and parent or carer report questionnaire data were used to derive these measures. Details about the risk thresholds can be found in Supplementary Material 1. Our focus in this research and more widely, is adolescent multiple risk behaviours [27, 38–40]. We chose these exposures a priori, owing to their known effect on cancer incidence and mortality and while the outcomes at age 24 are illustrative of longer-term trajectories, the real focus of this work is in identifying the patterns of risk behaviour across adolescence in order to identify intervention strategies. We therefore have not excluded any of these behaviours from the exposure measure because they are under-reported outcomes at age 24 years.

**Table 1:** Adolescent cancer risk behaviours and their derivation

Cancer risk behaviours	Definition/how derived			
	Age 11	Age 14	Age 16	Age 18
**Tobacco smoking**	Young person has ever smoked.	Young person has smoked cigarettes in past 6 months.	Young person smokes every week.	Young person smokes every week.
**Alcohol consumption**	Young person has had a whole drink before age 12 years.	Young person has had whole drink in past 6 months.	Young person has had 6 or more whole drinks in past 30 days.	Young person consumes alcohol ≥2–3 times a week or has hazardous alcohol consumption.
**Obesity**	Young person has a UK 1990 BMI population reference ≥95th centile.	Young person has a UK 1990 BMI population reference ≥95th centile.	Young person has a UK 1990 BMI population reference ≥95th centile.	Young person has a UK 1990 BMI population reference ≥95th centile.
**Sexual risk**	Young person has had penetrative sex without the use of a condom on the last occasion they had sex in the past year.	Young person has had penetrative sex without the use of a condom on the last occasion they had sex in the past year.	Young person has had penetrative sex without the use of a condom on the last occasion they had sex in the past year.	Young person has had penetrative sex without the use of a condom on the last occasion they had sex in the past year.
**Physical inactivity**	Young person has participated in vigorous physical activity 1–3 times a week or less (parent report).	Young person typically exercises < 5 times a week (self-report) or has participated in vigorous physical activity 1–3 times a week or less (parent report).	Young person typically exercises < 5 times a week (self-report) or has participated in vigorous physical activity 1–3 times a week or less (parent report).	Young person typically exercises < 5 times a week (self-report) or has participated in vigorous physical activity 1–3 times a week or less (parent report).

Sources of information:

T1/Age 11: data from sources when the participants were aged between 128 and 154 months, the midpoint of which is 141 months or 11.75 years

T2/Age 14: data from sources when the participants were aged between 166 and 171 months, the midpoint of which is 168.5 months or 14 years

T3/Age 16: data from sources when the participants were aged between 186 and 200 months, the midpoint of which is 193 months or 16 years

T4/Age 18: data from sources when the participants were aged between 214 and 224 months, the midpoint of which is 219 months or 18.25 years

### Outcome measures – early adult cancer risk

The early adult outcome measures are, where possible, more severe presentations of the adolescent cancer risk behaviours. For example, the adolescent smoking exposure ranges from ever smoked to weekly smoking, whereas the early adult outcome measures were daily smoking and having nicotine dependence. General obesity, as defined by height and weight, was supplemented by measures of central obesity: high waist circumference (≥80 cm for females and ≥ 94 cm for males, and high waist-hip ratio (≥0.85 for females and ≥ 1.00 for males) at age 24 years.

Early adult cancer risk was based on measurements collected in clinics (measured height and weight to compute body mass index, waist circumference and waist-hip ratio), or responses to questionnaires (harmful drinking, daily smoking and nicotine dependence) by participants at age ~ 24 years (mean age 24 years and 6 months, SD = 9.78 months). We were unable to include measures of accelerometery measured physical inactivity, owing to low numbers with a valid minimum number of days of wear-time (only 380 participants with 3 days of data). We were unable to estimate sexual risk using data about Chlamydia incidence because perfect prediction from measures integral to the final analysis was observed in the multiple imputation model, which may bias the relation of interest [41]. Binary indicators were derived for harmful drinking: a score of ≥8 in the Alcohol Use Disorders Identification Test-C (AUDIT-C); daily smoking; nicotine dependence (a score of ≥4 in the Fagerström test), obesity (a BMI of ≥30); high waist circumference, as defined by the National Institute of Health and Clinical Excellence (NICE) and World Health Organisation (WHO) guidelines: ≥80 cm (females) and ≥ 94 cm (males); and high waist-hip ratio (≥0.85 for females and ≥ 1.00 for males) [42, 43].

### Confounder measures

We identified potential confounders (common causes of both exposures and outcomes) that occurred before the exposure measures i.e. before age 11 years. All models were adjusted for: sex, intelligence quotient (IQ), parental socioeconomic status, adverse childhood experiences (ACEs), [44] maternal cannabis use, maternal harmful alcohol use, maternal smoking, child depressive symptoms (SMFQ), child total difficulties score (SDQ) and child antisocial behaviour. Models relating to the anthropometric outcomes (obesity, waist circumference and waist-hip ratio) were additionally adjusted for birthweight, gestational age, maternal obesity, maternal physical inactivity, and maternal unhealthy diet (see Supplementary Material 2 for more details of how confounder measures were derived).

### Statistical analysis

We summarised exposure to our adolescent cancer risk behaviours of interest (tobacco smoking, alcohol consumption, obesity, unprotected sexual intercourse, and physical inactivity) in two ways. We assigned each participant a score of one (risk present) or zero (risk not present) for each of the five risk behaviours at each of the four time points. Using the total risk score at each time point we then calculated a cumulative continuous score, summarising exposure to the five risk behaviours across adolescence and expressed the score as the area under the curve. This was done by summing the product of the total number of risks and the time interval, at four time points between ages ~ 11 and ~ 18 years (detailed explanations outlining the methods used to calculate this measure can be found in Supplementary Material 3). Second, using the same data, we derived longitudinal latent growth curves to explore whether the same behaviours cluster to produce qualitatively distinct risk profiles (over and above the cumulative continuous score). The processes used to derive the latent classes are described in more detail in Supplementary Material 4.

We explored the patterning of adolescent cancer risk behaviours, using quartiles of a continuous score summarising cumulative exposure to provide a comparative measure for the latent classes. We compared models with between 2 and 7 classes using both complete case and imputed data (see below for imputation method). The optimum model, as determined by the lowest Bayesian information criterion (BIC), was a 6-class latent class growth analysis, for both the imputation and complete case samples. These models produce a class-assignment probability indicating the confidence with which each participant can be allocated to a specific latent class. Entropy summarises this information as a single measure ranging from zero to one (one indicating absolute certainty that individuals have been assigned to the correct class). We have additionally provided analyses of the four-class solution in supplementary materials 7, 8 and 9, which despite having a higher BIC, provides a useful comparator for the quartiles.

Logistic regression analysis was used to examine prospective associations between quartiles of the continuous score and early adult cancer risk behaviours at age 24 years. We ran unadjusted models for all outcomes, including only the exposure and outcome measures followed by a sequence of adjusted models, which additionally controlled for: (i) partially adjusted: sex, IQ, parental socioeconomic status and adverse childhood experiences (ACEs); and (ii) fully adjusted: maternal cannabis use, maternal harmful alcohol use, maternal smoking, child depressive symptoms (SMFQ), child total difficulties score (SDQ) and child antisocial behaviour. Models for obesity, waist circumference, and waist-hip ratio outcomes were additionally adjusted for birthweight, gestational age, maternal obesity, maternal physical inactivity, and maternal unhealthy diet.

### Missing data

Data on all exposures at one time point were available for 6351 (46.0%) of ALSPAC participants. As shown in Table 2, among this sample, data were complete for the sex variable, near complete for the confounder measures (for example, housing tenure *n* = 6169, 97.1%); available for ~ 50% of the sample for the outcome measures (obesity *n* = 3202, 50.4%); and available for 1951 (30.7%) for the exposure measure. In our primary analysis, multiple imputation was used to account for missing data (see below). In sensitivity analyses, we investigated associations for each 24-year outcome on complete case samples i.e. those with no missing data on any of exposure, outcome or confounder measures. The flow diagram for deriving the sample can be found in Supplementary Material 5. The subsample of ALSPAC participants selected for imputation is not a random sample: they are more likely to be female and less likely to be from the lowest income quintile; to be living in privately or subsidised rental property; to have a mother with fewer educational qualifications and have lower parental social class (*p* < 0.001). Please see Supplementary Material 6 which provides a comparison of the imputation sample and those excluded from the analysis by key demographic variables, where we also provide a detailed discussion about why we do not think this leads to selection bias. Multivariate imputation by chained equations was carried out using the ‘ice’ routine in Stata. This approach is based on the missing at random (MAR) assumption, i.e. that any differences between the missing and observed values, can be explained by differences in the observed data [45]. All variables used in the analyses, including the outcome measures, exposure measures and confounders were included in the imputation model, along with alternative measures that had been collected at different times. These were included as auxiliary variables to reduce bias by improving the precision of the imputation model. Monte Carlo errors were used to compare the results obtained when imputing 25, 100 and 200 data sets. Imputed results shown have been pooled across the 200 data sets, having satisfied White et al.’s rules of thumb for the number of imputations [46]. All analysis was conducted using Stata version 15 [47] and Mplus version 8 [48].

**Table 2:** Sample descriptive statistics^a^

Outcome measures	Available case sample by variable	Complete case sample 1951	Imputation sample 6351
		n (%)	% (SE for the percentage)
Harmful alcohol (Yes)	3167	273 (18.9%)	16% (0.6)
No		1169 (81.1%)	84% (0.6)
Daily smoking (Yes)	3195	121 (8.3%)	14% (0.6)
No		1330 (91.7%)	86% (0.6)
Nicotine dependence (Yes)	3193	50 (3.5%)	6% (0.4)
No		1400 (96.6%)	94% (0.4)
Obesity (Yes)	3202	183 (12.6%)	11% (0.5)
No		1270 (88%)	89% (0.5)
High waist circumference (Yes)	3194	411 (28.3%)	29% (0.7)
No		1042 (71.7%)	71% (0.7)
High waist-hip ratio (Yes)	3194	114 (7.9%)	10% (0.4)
No		1339 (92.2%)	90% (0.4)
**Exposure measure**			
Cancer Risk Exposure AUC (0–21)	1951		
Mean (SD)		9.57 (4.21)	9.72 (3.98)
Median (IQR)		8.75 (6.5–12)	9.63 (6.6–12.1)
**Confounder measures**			
Sex	6351		
Female		1171 (60%)	52% (0.6)
Male		780 (40%)	48% (0.6)
Maternal education	5540		
Degree		458 (24%)	17% (0.5)
A level		562 (29%)	28% (0.6
O level		632 (33%)	35% (0.6)
< O level		271 (14%)	20% (0.5)
Parental socioeconomic position	5319		
Professional		401 (21%)	17% (0.5)
Managerial and technical		886 (47%)	45% (0.7)
Skilled non-man		417 (22%)	24% (0.6)
Skilled man, part or unskilled		169 (9%)	14% (0.5)
Housing tenure	6169		
Mortgage/owned		1704 (89%)	84% (0.5)
Private rent		91 (5%)	8% (0.4)
Subsidised rent		127 (7%)	8% (0.3)
Income	5105		
High		490 (27%)	24% (0.6)
Mid-high		454 (25%)	22% (0.6)
Middle		394 (22%)	21% (0.6)
Mid-low		305 (17%)	19% (0.6)
Low		183 (10%)	15% (0.5)
IQ: mean (SD)	5433	109.8 (16.18)	105.4 (17.53)
Maternal smoking	5342	218 (12%)	18% (0.5)
No		1632 (88%)	82% (0.5)
Harmful maternal alcohol consumption	5170	481 (27%)	25% (0.6)
No		1321 (73%)	75% (0.6)
Maternal cannabis use	5377	58 (3%)	4% (0.3)
No		1798 (97%)	96% (0.3)
Maternal physical activity (hrs per week): mean (SD)	5604	3.35 (5.38)	Mean = 3.96 (6.38)
Birthweight: mean (SD)	5347	3437.5 (507.6)	Mean = 3440 (559.45)
Gestational age: mean (SD)	5347	276.8 (11.9)	Mean = 276.2 (13.55)
Maternal unhealthy diet: mean (SD)	5201	0.03 (0.79)	Mean = 0.08 (0.80)
Child depressive symptoms short moods and feelings questionnaire (SMFQ): mean (SD)	5347	2.27 (2.93)	Mean = 2.47 (3.19)
Child antisocial behaviour: mean (SD)	5276	0.26 (0.68)	Mean = 0.34 (0.80)
Child total difficulties score strengths and difficulties questionnaire (SDQ): mean (SD)	5459	6.00 (4.42)	Mean = 6.58 (4.78)
Adverse childhood experiences (ACEs): mean (SD)	3396	1.03 (1.21)	Mean = 1.31 (1.59)
Maternal Body mass index (BMI): mean (SD)	5749	22.59 (3.52)	Mean = 22.88 (3.98)

^a^To calculate the number of cases missing in the imputation sample, subtract the available case sample by variable from 6351

*SD* standard deviation, *IQR* inter quartile range, *SE* standard error

## Results

Table 2 outlines the descriptive statistics for the complete case sample and the imputed data sample used in this research. For the complete case sample, high waist circumference with a prevalence of 411 (28.3%) was the most frequently occurring outcome at age 24 years. This was followed by harmful alcohol 273 (18.9%), obesity 183 (12.6%), daily smoking 121 (8.3%), high waist-to-hip ratio 114 (7.9%), and nicotine dependence 50 (3.5%).

### Patterning of adolescent cancer risk behaviours

**Fig. 1:**
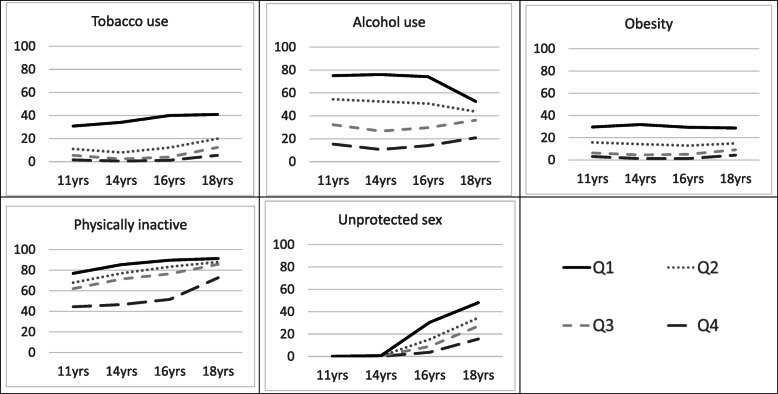
Cumulative risk score by quartiles and separate behaviours across adolescence, line graphs showing each quartile of risk against each risk behaviour across adolescence

Examination of the quartiles of a continuous score summarising cumulative exposure to adolescent cancer risk shown in Fig. 1, revealed that adolescents in the top 25% of participants, (Q1: shown with the solid black line) consistently had the highest chance of exhibiting all adolescent risk behaviours at each of the four time points, indicating that there is a group of young people who consistently undertake multiple cancer risk behaviours across adolescence. There were six latent classes, which are described in more detail in Supplementary Materials 7 and 8. We found that quartiles of cumulative exposure were highly consistent with the latent classes. For example, 100% of those in the persistent very low-risk class belonged to the lowest quartile of the cumulative exposure measure, and vice versa, where 20% of those in the lowest quartile belonged to the persistent very low-risk class and 80% to the persistent low-risk class (see Supplementary Material 9).

However, while the quartiles and the latent classes were quantitatively similar, there were some qualitative differences (see Supplementary Material 7). For example, although the persistent-low and low-to-increasing risk classes had similar average cumulative risk scores, their risk profiles were different. Adolescents in the persistent low-risk class had consistently low-risk of all behaviours throughout adolescence, whereas those in the low-increasing-risk class had a rapidly increasing risk of alcohol use, physical inactivity, and sexual risk from age 14 years. Similarly, the moderate-increasing-risk and persistent-moderate-risk classes had similar average cumulative risk scores. However, while their obesity and physical inactivity risk was similar across adolescence, the moderate-increasing-risk class had increasing risk of tobacco and alcohol use and sexual risk, while the persistent-moderate-risk class had low cumulative tobacco use and sexual risk and decreasing alcohol use (a similar analysis using the 4-class solution can also be found in supplementary materials 7, 8 and 9). Unfortunately, entropy for the latent class results was poor (6-class solution: 0.64 for complete case and 0.53 for the imputed sample and 4-class solution: 0.61 for complete case and 0.51 for the imputed sample), meaning that around one third of participants have an incorrect class assignment for the complete case sample and just under half have an incorrect assignment for the imputed sample.

### Social patterning of the exposure and outcome measures

There was evidence that the quartiles of adolescent cancer risk were socially patterned by both maternal education and parental social class (see Supplementary Material 10). However, the effect for income was non-monotonic and those in the middle-low income group, as opposed to the low-income group, had the highest adolescent risk scores.

There were some differences in the prevalence of the 24-year outcomes comparing males with females (Table 3). Males had increased odds of harmful drinking at age 24 years compared with females, (odds ratio (OR) = 1.68; 95%CI:1.42 to 1.99). However, males had decreased odds of all the anthropometric measures compared to females: obesity (OR = 0.72; 95%CI:0.57 to 0.89), high waist circumference (OR = 0.44; 95%CI:0.37 to 0.53) and high waist-hip ratio: (OR = 0.18 95%CI:0.12 to 0.27). There were no differences between males and females with respect to odds of either smoking outcomes. There was social patterning of the outcomes, when compared to the reference category (in each case the highest social class, level of maternal education, or income quintile). The odds of all outcomes increased for every incremental decrease in social position. There was one notable exception, harmful drinking, where the social patterning ran the opposite direction and for every incremental increase in social position, there were increased odds of harmful drinking.

**Table 3:** Social patterning of outcomes at age 24 years (imputed data)

	Harmful drinking	Daily smoking	Nicotine dependence	Obesity	Waist circumference	Waist-hip ratio
	Odds ratio [95% CI]	Odds ratio [95% CI]	Odds ratio [95% CI]	Odds ratio [95% CI]	Odds ratio [95% CI]	Odds ratio [95% CI]
*Sex*						
Female (ref)	1	1	1	1	1	1
Male	1.68 [1.42,1.99]	1.01 [0.81,1.26]	1.08 [0.79,1.48]	0.72 [0.57,0.89]	0.44 [0.37,0.53]	0.18 [0.12,0.27]
*Household income*						
High (ref)	1	1	1	1	1	1
Middle high	0.93 [0.74,1.17]	1.13 [0.80,1.60]	1.03 [0.62,1.71]	0.96 [0.68,1.34]	1.31 [1.03,1.67]	1.17 [0.75,1.83]
Middle	0.83 [0.64,1.08]	1.24 [0.87,1.76]	1.45 [0.88,2.37]	1.21 [0.86,1.70]	1.55 [1.21,1.99]	1.60 [1.03,2.49]
Middle low	0.61 [0.46,0.81]	1.39 [0.97,1.99]	1.22 [0.72,2.08]	1.77 [1.27,2.45]	1.69 [1.31,2.18]	1.54 [0.97,2.44]
Low	0.65 [0.48,0.89]	1.97 [1.36,2.86]	1.55 [0.89,2.71]	1.70 [1.18,2.46]	1.77 [1.33,2.34]	2.40 [1.51,3.81]
*Maternal educational attainment*						
Degree (ref)	1	1	1	1	1	1
A level	0.79 [0.63,1.00]	1.23 [0.86,1.74]	1.21 [0.70,2.06]	1.49 [1.04,2.12]	1.22 [0.96,1.54]	1.35 [0.86,2.13]
O level	0.64 [0.51,0.80]	1.57 [1.12,2.18]	1.88 [1.14,3.08]	2.00 [1.43,2.81]	1.57 [1.25,1.98]	2.00 [1.31,3.07]
< O level	0.50 [0.37,0.67]	2.03 [1.40,2.94]	2.27 [1.31,3.93]	2.72 [1.88,3.94]	2.33 [1.79,3.03]	2.57 [1.61,4.08]
*Parental social class*						
Professional (ref)	1	1	1	1	1	1
Managerial and technical	0.94 [0.75,1.18]	1.82 [1.26,2.64]	1.69 [0.96,2.96]	1.69 [1.19,2.40]	1.44 [1.14,1.81]	1.59 [1.03,2.45]
Skilled non-manual	0.70 [0.53,0.91]	2.35 [1.58,3.48]	2.53 [1.41,4.53]	2.24 [1.55,3.25]	1.61 [1.25,2.08]	1.88 [1.18,2.99]
Skilled manual, etc.	0.66 [0.47,0.93]	3.31 [2.14,5.11]	3.53 [1.88,6.63]	2.91 [1.92,4.43]	2.41 [1.78,3.25]	2.46 [1.46,4.14]

### Associations between multiple adolescent cancer risk behaviours and at age 24 years

Associations of large magnitude were present between adolescent and early adult cancer risk behaviours (see Table 4). When compared to the bottom quartile, those in the top quartile had more than five times greater odds of nicotine dependency at age 24 years: (odds ratio, OR = 5.37; 95% confidence interval, CI: 3.64 to 7.93) and daily smoking (OR = 5.10; 95%CI: 3.19 to 8.17); nearly five times the odds of being obese (OR = 4.84; 95%CI:3.33 to 7.03); nearly two and a half times the odds of a high waist circumference (OR = 2.48 95%CI:1.94 to 3.16); just more than twice the odds of harmful drinking (OR = 2.04 95%CI: 1.57 to 2.65); and nearly twice the odds of a high waist-hip ratio (OR = 1.88 95%CI:1.30 to 2.71).

**Table 4:** Odds ratios [95% confidence intervals] for cancer risk behaviours at age 24 years (imputed sample)

Harmful drinking	Unadjusted analysis	*p*-value	Partially adjusted analysis^a^	*p*-value	Fully adjusted analysis^b^	*p*-value
	Odds ratios [95% CIs]		Odds ratios [95% CIs]		Odds ratios [95% CIs]	
Q1 (referent category)	1	*p* ≤ 0.001	1	*p* ≤ 0.001	1	*p* ≤ 0.001
Q2	1.20 [0.92,1.55]		1.27 [0.98,1.65]		1.29 [0.99,1.68]	
Q3	1.54 [1.21,1.96]		1.69 [1.32,2.17]		1.71 [1.33,2.19]	
Q4	1.71 [1.33,2.18]		2.03 [1.57,2.62]		2.04 [1.57,2.65]	
Linear association	1.04 [1.03,1.06]	*p* ≤ 0.001	1.06 [1.04,1.08]	*p* ≤ 0.001	1.06 [1.04,1.08]	*p* ≤ 0.001
**Daily smoking**						
Q1 (referent category)	1	*p* ≤ 0.001	1	*p* ≤ 0.001	1	*p* ≤ 0.001
Q2	1.69 [1.14,2.51]		1.61 [1.08,2.40]		1.57 [1.05,2.3]	
Q3	2.73 [1.86,3.99]		2.57 [1.75,3.79]		2.50[1.69,3.70]	
Q4	5.89 [4.04,8.58]		5.71 [3.90,8.38]		5.37[3.64,7.93]	
Linear association	1.18 [1.15,1.22]	*p* ≤ 0.001	1.18 [1.15,1.22]	*p* ≤ 0.001	1.18[1.15,1.21]	*p* ≤ 0.001
**Nicotine dependence**						
Q1 (referent category)	1	*p* ≤ 0.001	1	*p* ≤ 0.001	1	*p* ≤ 0.001
Q2	1.50 [0.87,2.58]		1.42 [0.82,2.46]		1.37 [0.79,2.38]	
Q3	2.54 [1.57,4.12]		2.36 [1.46,3.84]		2.26 [1.39,3.69]	
Q4	5.93 [3.75,9.36]		5.61 [3.53,8.93]		5.10 [3.19,8.17]	
Linear association	1.19 [1.15, 1.23]	*p* ≤ 0.001	1.18 1.14, 1.23]	*p* ≤ 0.001	1.18 [1.13, 1.22]	*p* ≤ 0.001
**Obesity**^**c**^						
Q1 (referent category)	1	*p* ≤ 0.001	1	*p* ≤ 0.001	1	*p* ≤ 0.001
Q2	1.54 [1.05,2.28]		1.48 [1.00,2.19]		1.52 [0.98,2.34]	
Q3	2.70 [1.89,3.84]		2.53 1.77,3.61]		2.41 [1.62,3.58]	
Q4	5.74 [4.12,8.00]		5.26 [3.76,7.37]		4.84 [3.33,7.03]	
Linear association	1.18 [1.15, 1.21]	*p* ≤ 0.001	1.17 [1.14, 1.20]	*p* ≤ 0.001	1.16 [1.13, 1.19]	*p* ≤ 0.001
**Waist circumference**^**c**^						
Q1 (referent category)	1	*p* ≤ 0.001	1	*p* ≤ 0.001	1	*p* ≤ 0.001
Q2	1.34 [1.08,1.68]		1.25 1.00,1.57]		1.24 [0.97,1.59]	
Q3	1.77 [1.43,2.19]		1.59 [1.28,1.98]		1.53 [1.21,1.94]	
Q4	3.17 [2.55,3.93]		2.66 [2.13,3.32]		2.48 [1.94,3.16]	
Linear association	1.12 [1.10,1.14]	*p* ≤ 0.001	1.10 [1.08, 1.12]	*p* ≤ 0.001	1.09 [1.07, 1.12]	*p* ≤ 0.001
**Waist-to-hip ratio**^**c**^						
Q1 (referent category)	1	*p* ≤ 0.001	1	*p* ≤ 0.001	1	*p* ≤ 0.001
Q2	1.47 1.04,2.10]		1.27 [0.88,1.83]		1.29 [0.87,1.91]	
Q3	1.72 [1.23,2.40]		1.37 [0.97,1.95]		1.32 [0.90,1.94]	
Q4	2.87 [2.09,3.93]		1.98 [1.42,2.78]		1.88 [1.30,2.71]	
Linear association	1.10 [1.07, 1.13]	*p* ≤ 0.001	1.07 [1.04, 1.09]	*p* ≤ 0.001	1.06 [1.03, 1.09]	*p* ≤ 0.001

^a^ Adjusted for sex, IQ, parental socio-economic status (maternal education, parental social class, household equivalised income and housing tenure) and adverse childhood experiences (ACEs)

^b^ Additionally adjusted for maternal cannabis use, maternal harmful alcohol use and maternal smoking and child depressive symptoms (SMFQ), child antisocial behaviour and child total difficulties score (SDQ)

^c^ These analyses were additionally, adjusted for birthweight and gestational age in the partially adjusted analysis and maternal obesity, maternal physical inactivity and maternal unhealthy diet in the fully adjusted analysis

**Fig. 2:**
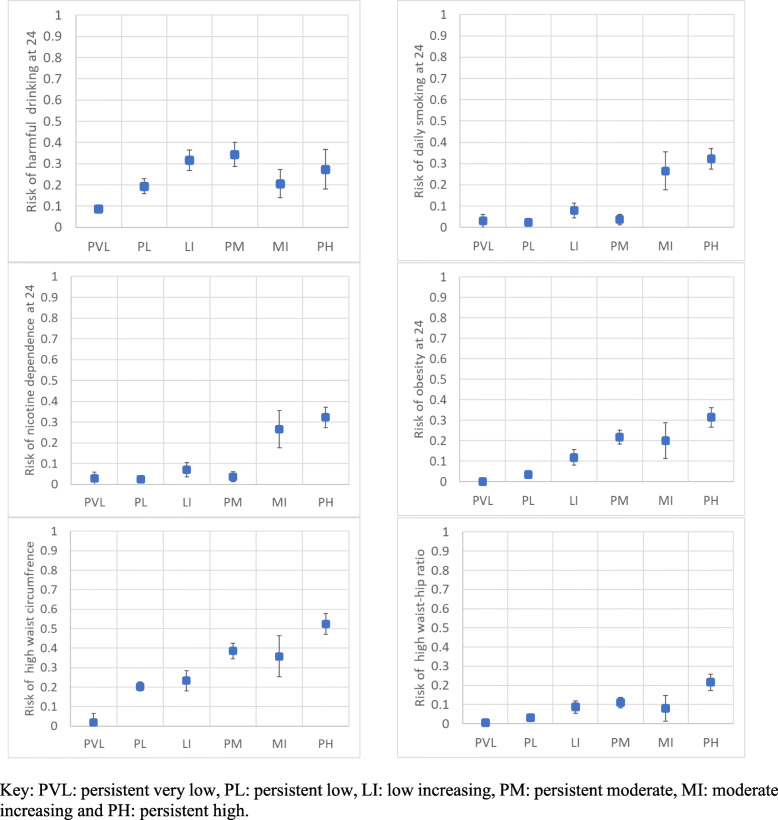
Latent classes (6-class solution) and risk of early adult cancer behaviours at age 24 years, box and whisker charts showing each of the 6 latent classes against each of the outcomes at age 24

**Fig. 3:**
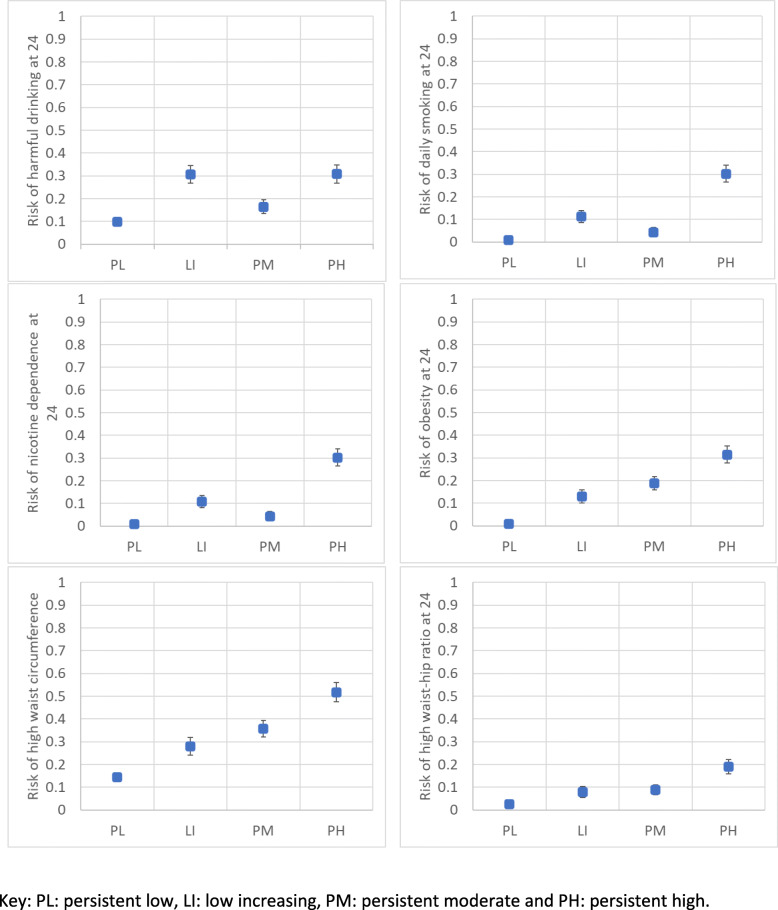
Latent classes (4-class solution) and risk of early adult cancer behaviours at age 24 years, box and whisker charts showing each of the 4 latent classes against each of the outcomes at age 24

With reference to Fig. 2 the 6-class solution, the persistent very low-risk and persistent low-risk classes in adolescence consistently had the lowest risk of all outcomes at age 24 years. The persistent high-risk class in adolescence consistently had the highest risk of all outcomes at age 24 years, except harmful drinking, which had a non-monotonic association with the adolescent latent classes (low-increasing- and persistent-moderate-risk classes in adolescence had the highest risk of harmful drinking at 24). The moderate-increasing- and persistent-high-risk classes in adolescence had the highest risk of both tobacco outcomes at age 24. Finally, the anthropometric outcomes (obesity, high waist circumference and high waist-hip ratio) largely followed a linear association between latent class in adolescence and risk of each outcome at age 24. With reference to Fig. 3, the results for the 4-class solution are very similar and provide a useful comparator for the quartiles. The persistent high-risk class consistently had the highest risk of all outcomes at age 24 years, except harmful drinking, which had a non-monotonic association with the adolescent latent classes (the low-increasing risk class had the same risk of harmful drinking at 24). Similarly, the anthropometric outcomes (obesity, high waist circumference and high waist-hip ratio) largely followed a linear association between latent class in adolescence and risk of each outcome at age 24.

## Discussion

Using repeated measures of cancer risk behaviours at four occasions across adolescence, we used two different methods to derive patterns of adolescent cancer risk. We found distinct groups of adolescents characterised by consistently high- and low-cancer risk behaviours during adolescence. We also found associations of large magnitude between adolescent and early adult cancer risk behaviours. We demonstrate for the first time that multiple cancer risk behaviours pattern longitudinally across adolescence and into young adulthood. Unlike previous research which focusses on either multiple risk behaviours at one time point (using cross-sectional data), or longer-term trajectories of single risk behaviours, our research combines the two. We have included a multiplicity of risk behaviours (five separate risk behaviours), using longitudinal data (at four different time points) covering a minimum 13-year period. Preventing multiple cancer risk behaviours during adolescence would likely reduce these behaviours in early adulthood and across the life-course, thereby reducing cancer incidence and mortality.

The strengths of this study were the long-term, longitudinal design. We were able to show associations between an exposure measure that covered the whole adolescent period (age 11–18 years) and outcomes 6 years later at age ~ 24 years. This has the advantage that most young people will have left education and entered the labour market and therefore our findings are indicative of a longer-term trajectory across different environments. We used both a cumulative score and latent classes to capture the exposure measure, which provided different insights, but were mutually reinforcing. We were also able to adjust for an extensive range of potential confounders, reducing the chance of residual confounding.

The weaknesses of the study include the potential for residual confounding and cohort attrition, as in all observational studies. However, for selection bias to pose a problem, our outcome measures would have to be conditionally related to whether a participant remains in the sample or missing not at random (MNAR) [45, 49]. We assume that our data is missing at random (MAR). Many of the measures were collected using self-report, which might mean participants favour responses they believe are more socially acceptable rather than choosing responses that reflect their true feelings. However, by the time ALSPAC participants had reached adolescence, following years of completing research questionnaires, it is likely that they will have developed trust in the maintenance of their anonymity, possibly reducing such bias. The risk behaviours were all reduced to binary variables in order to calculate the score total, which may lead to a loss of information. However, in line with our previous research and given the highly differential risks associated with these behaviours, at different levels and for different cancers, each risk has an equal weighting, as it would not be possible to accurately weight the risk behaviours differently. We were unable to include unhealthy diet as part of the adolescent exposure, as it was not collected in ALSPAC past the age of 13 years. We were also unable to include outcome measures relating to physical inactivity or sexual risk, owing to the extent of their missingness and the problem of perfect prediction as outlined in the methods. With reference to the latent class analysis, entropy was poor (6-class solution: 0.64 for complete case and 0.53 for the imputed sample and 4-class solution: 0.61 for complete case and 0.52 for the imputed sample). Therefore, while the classes may provide further insight into the patterns of adolescent behaviours, we cannot be confident that participants have been separated into the correct classes, which limits their utility as a targeting tool when considering public health interventions. They are also difficult to replicate because we would require the same number of similar exposures, at similar time points, which is not always available in other data sets. Finally, ALSPAC is not a nationally representative sample and therefore may limit the generalisability of our findings.

Given the co-occurrence of these behaviours and their associations with early adult risk behaviours, public health policies should adopt approaches that enable all health professionals who have contact with adolescents - including sexual health clinicians, general practitioners (GPs), public health workers and policy makers - to address a multitude of risks, at each contact. As a third of the cohort were identified to be persistently-moderate, or persistently-high engagers in risk throughout adolescence, it may be that early intervention is required to prevent engagement at an early stage in adolescence.

We found evidence that both the adolescent exposure measure and the outcomes at age 24 years (except harmful drinking) were socially patterned, i.e. the likelihood/odds of exposure to these risk behaviours increased for every incremental decrease in social position. However, interventions that focus on the most disadvantaged people, will not redress the social gradient and will only tackle a small part of the problem, while targeting subgroups excludes other deprived groups and risks stigmatising those targeted [50]. Further, because the social patterning for harmful drinking, ran in the opposite direction, adopting this strategy would be ineffective if we targeted interventions in this way. Universal interventions aimed at behaviour change at the individual level, have been shown to further exacerbate health inequalities, because more advantaged people and those who already have healthy behaviours tend to be quicker and more likely to take up these types of interventions [51]. Given this, and the social gradient of our exposure and outcome measures, interventions that are universal, but with a scale and intensity that is proportionate to the level of risk, [50] are preferable. Previous research relating to the social patterning of alcohol consumption has been mixed. For example, Melotti and colleagues found that adolescents that come from higher-income households in childhood were more likely to use alcohol. However, those with mothers with more educational qualifications were less likely to use alcohol [52]. Possible explanations that young adults with higher socioeconomic status in ALSPAC are more likely to be harmful drinking, include having increased disposable income available to them, which facilitates the purchase of alcohol, not available to less well-off individuals. An alternative explanation is provided by Luthar [53] who argues that young people from affluent families feel more pressure to achieve and are more isolated from their parents which contributes to their unhealthy behaviours. It is also possible that young people from higher socioeconomic backgrounds who are more likely to attend higher education, maintain drinking habits they acquired at university, into their early twenties.

A recent Cochrane systematic review showed that applying the World Health Organisation (WHO) health promoting schools framework improves some aspects of student health, including body mass index (BMI), physical activity, physical fitness and tobacco use, to an extent that is important at the population level. However, it found little evidence for improvement in zBMI (BMI, standardized for age and gender), and no evidence for alcohol use [54]. Another Cochrane Systematic Review looking at individual-, family-, and school-level interventions targeting multiple risk behaviours in young people, demonstrated that universal school-based interventions are most effective in preventing adolescent alcohol use, tobacco smoking, illicit drug use and antisocial behaviour, and increasing physical activity, but did not find strong evidence of benefit for family or individual-level interventions [55]. Therefore, efforts at preventing these behaviours should focus on developing appropriate proportionate universalist interventions in secondary schools.

We have shown that a multitude of behaviours known to cause cancer (tobacco smoking, alcohol consumption, obesity, unprotected sexual intercourse, and physical inactivity), originate in adolescence. Future research should focus on identifying the antecedents of adolescent cancer risk behaviours to identify opportunities for earlier intervention before the behaviours begin. Research is also required into whether the associations observed in ALSPAC are evident in similar cohort studies and outside of the UK.

## Conclusions

We have shown that exposure to adolescent cancer risk behaviours greatly increased the odds of cancer risk behaviours in early adulthood. Interventions to reduce these behaviours should target multiple rather than single risk behaviours and should focus on adolescence. Given this, and the social gradient of our exposure and outcome measures, school-based interventions that are universal, but with a scale and intensity that is proportionate to the level of risk are preferable.

## Supplementary Information

**Additional file 1.** Supplementary Material, Description of data: The supplementary material contains additional information concerning: 1. Risk thresholds; 2. Confounder measures and their derivation; 3.Deriving the adolescent cumulative risk exposure measure; 4. Deriving the latent classes; 5. Deriving the sample; 6. Comparison of imputation sample and those excluded from the analysis by key demographic variables; 7.Risk profiles for adolescence, percentage of sample and cumulative risk score for complete case and imputed samples; 8. Latent class trajectories; 9. Cross tabulations of cumulative risk score quartiles and proportion belonging to each of the latent classes; and 10. Associations between the adolescent cancer risk measure and socioeconomic status (SES) measures.

**Additional file 2.** STROBE Statement—Checklist of items that should be included in reports of cohort studies, Description of data: a table providing information regarding where each of the components of the report are located in the document.

**Additional file 3.** Data sources. Description of data: contains both a table showing the sources of ALSPAC questionnaire and clinic data used in research and links to each questionnaire or data source.

## Data Availability

The datasets supporting the conclusions of this article are available from ALSPAC. The ALSPAC policy on data sharing is available at www.bristol.ac.uk/alspac. To discuss access to ALSPAC data, please contact the ALSPAC executive team on alspac-exec@bristol.ac.uk. Details of all available questionnaires can be found using the following link (http://www.bristol.ac.uk/alspac/researchers/our-data/questionnaires/child-completed-questionnaires/). We have used several questionnaires, the details of which can be found in the supplementary materials file, Data sources.

## Abbreviations

ALSPAC: Avon Longitudinal Study of Parents and Children
BMI: Body mass index
