# Supplementary material for: Young adult cancer risk behaviours originate in adolescence: a longitudinal analysis using ALSPAC, a UK birth cohort study
Source: BMC Cancer. 2021 Apr 7;21:365. doi: 10.1186/s12885-021-08098-8 (PMC8028717; doi:10.1186/s12885-021-08098-8)
Supplement: Supplementary file 1 — Additional file 1. Supplementary Material, Description of data: The supplementary material contains additional information concerning: 1. Risk thresholds; 2. Confounder measures and their derivation; 3.Deriving the adolescent cumulative risk exposure measure; 4. Deriving the latent classes; 5. Deriving the sample; 6. Comparison of imputation sample and those excluded from the analysis by key demographic variables; 7.Risk profiles for adolescence, percentage of sample and cumulative risk score for complete case and imputed samples; 8. Latent class trajectories; 9. Cross tabulations of cumulative risk score quartiles and proportion belonging to each of the latent classes; and 10. Associations between the adolescent cancer risk measure and socioeconomic status (SES) measures. [file 12885_2021_8098_MOESM1_ESM.docx]

# Supplementary material

The supplementary material contains additional information concerning:

1. Risk thresholds
2. Confounder measures and their derivation
3. Deriving the adolescent cumulative risk exposure measure
4. Deriving the latent classes
5. Deriving the sample
6. Comparison of imputation sample and those excluded from the analysis by key demographic variables
7. Risk profiles for adolescence, percentage of sample and cumulative risk score for complete case and imputed samples
8. Latent class trajectories
9. Cross tabulations of cumulative risk score quartiles and proportion belonging to each of the latent classes
10. Associations between the adolescent cancer risk measure and socioeconomic status (SES) measures

## Risk thresholds

Decisions regarding the adolescent and adult risk thresholds were informed by evidence from the World Cancer Research Fund (WCRF) Network’s Continuous Update Project (CUP) (1) (WCRF CUP 2018), evidence from the International Agency for Research on Cancer (IARC) (2) and national guidelines on cancer risk behaviours. We also used our knowledge of adolescent multiple risk behaviours and tried to reflect where possible, behaviours that are age appropriate.

The Continuous Update Project (CUP) is an ongoing programme conducted by the WCRF that analyses cancer prevention and survival research related to alcoholic drinks, body fatness and weight gain, diet, nutrition and physical activity and sedentary behaviour. Research is continually added to the CUP’s database and systematically reviewed by a team at Imperial College London. An independent panel of experts carries out ongoing evaluations of this evidence, and their findings form the basis of their recommendations. This document was published in 2018 and is an update from an equivalent report from 2007. We have only included exposures from the report which are deemed to have either convincing or probable evidence (as opposed to limited evidence) of an increased or decreased risk of cancer.

### Tobacco smoking

Any exposure to tobacco smoking is a cancer risk and increases with duration of smoking and number of cigarettes smoked, smoking cessation reduces smoking risk, but the risks never drop to that of never smokers (2). However, given the longitudinal nature of the research we cannot use ever smoked as a threshold after the first time point. We were also wary of classifying an adolescent as a smoker if they had only tried cigarettes once. We therefore used: smoked cigarettes in the past 6 months for the second measure; and to be consistent with the NHS in the UK which defines regular smokers as “usually smoking at least one cigarette per week” and our previous research relating to multiple risk behaviours, (3–6) we used the threshold smokes every week for the third and fourth measures at age ~16 and ~18 years, respectively.

### Alcohol consumption

Any exposure to alcohol is a cancer risk, however, we felt it was important to distinguish between young people who were merely trying alcohol (characterised as sipping), from young people who have had whole alcoholic drinks - a whole drink is categorised as a whole can of beer, glass of wine, measure of spirits, bottle of alcopop and is not just sips of alcohol. We therefore categorised those who have had a whole drink by age 12 years as the first measure and those who have had a whole drink in the past 6 months as the second measure. While the CUP provides evidence of a dose-response relationship with alcohol, they do not provide definitive thresholds, so we used the UK’s national guidance. In the UK, national guidelines at the time were that children and young people are advised not to drink alcohol before age 18 years. However, if children do drink alcohol underage, it should not be until they are at least 15. If 15 to 17-year olds drink alcohol, it should be rarely, and never more than once a week. We have tried to reflect this and the increasing normativity of alcohol consumption for the latter two measures to those who drank 6 or more whole drinks in past 30 days as the third measure; and consumes alcohol 2-3 times a week or more or hazardous drinking, as the final measure.

### Obesity

There is strong evidence that being obese throughout adulthood increases the risk of mouth, pharynx and larynx, oesophagus, stomach, pancreas, gallbladder, liver, colorectum, post menopause breast, ovary, endometrium, prostate and kidney cancer. These associations generally follow a dose–response relationship, so there is also a smaller increase in risk for people who are overweight but not obese (1). Body fatness is difficult to measure directly, however, because it is the most changeable determinant of weight, various markers of body fatness have been developed – body mass index (BMI) being the most common. Each of the measures of obesity (UK 1990, WHO 2007 IOTF) have advantages and disadvantages, so any decision will be pragmatic. We have therefore chosen to use all three measures, the UK 1990, as that is what has predominantly been used in previous ALSPAC analysis.

### Unprotected sexual intercourse

Human papilloma viruses (HPV) are transmitted during unprotected sexual intercourse. HPV ‘is a necessary cause of cervical cancer [and] its genome can be found in nearly all cervical cancers’ (2). It is generally accepted that 100% of cervical cancers are attributable to 13 high risk HPV types classified as carcinogenic or probably carcinogenic. HPV16 and 18 are the most prevalent types and account for 70% of cervical cancer in the all world regions. Head and neck cancers are a large and heterogeneous group of malignancies, and tobacco smoking and alcohol consumption are the major causes. However, HPV DNA has been reported to be present in a variable fraction of head and neck cancers (0–60%). Among HPV-positive cancers, HPV16 predominates (90% of HPV-positive carcinomas). HPV prevalence is notably high in oropharyngeal cancer, in which HPV16 is present at, localized to cell nuclei, frequently integrated into the cell genome, and actively transcribing viral oncoproteins E6 and E7 (2).

### Physical inactivity

Evidence relating to physical activity in the WCRF’s CUP reflects the lack of a generally agreed upon classification for what constitutes physical activity. In highest verses lowest analyses, physical activity relates to metabolic equivalents or MET hours per week or a MET score. The highest verses lowest physical activity in the individual studies were generally defined as ‘active’ and ‘inactive’, respectively and there were wide between-study variations in how activity was defined. Other measures used included: the quantity of physical activity per day/week, as well as more qualitative measures. There was only ‘limited’ evidence that sedentary behaviour is associated with increased risk of cancer, so this has not been included in our analyses. Owing to a lack of definitive thresholds in the CUP, our threshold was informed by UK national guidelines and by what was available in the ALSPAC data, i.e. the young person typically exercises <5 times a week or has participated in vigorous physical activity 1-3 times a week or less.

### Adult measures

We wanted the adult measures where possible, to reflect more acute presentations of the adolescent cancer risk behaviours. We therefore chose daily smoking, nicotine dependence, harmful drinking, obesity, high waist circumference and high waist-hip ratio.

## Confounder measures and their derivation

**Sex**: biological sex recorded at birth.

**Parental socioeconomic status**: measured using parent’s highest social class (professional; managerial and technical; skilled non-manual; and skilled manual, part or unskilled manual), mother’s highest educational level (degree, A-level, O-level/GCSE and less than O-level/GCSE), household income (divided into quintiles of high to low income), housing tenure during pregnancy (mortgaged or own property, privately rented property or subsidised rental property).

**Adverse childhood experiences (ACEs):** mothers, partners and the study child were asked 288 questions over 27 data collection points about the child’s exposure to nine ACEs up to age 9 years. ACEs include child sexual, physical, or emotional abuse; parent substance use; parent mental health problems or suicide attempt; violence between parents; parental separation; parental criminal conviction; and child bullying. ACEs were derived as in previous papers (7,8).

**IQ:** The Wechsler Intelligence Scale for Children (WISC) intelligence quotient (IQ) test was used to assess cognitive function at age 8 years (9).

**Antisocial behaviour:** measured using the self-reported antisocial behaviour for young children questionnaire was used to produce an antisocial behaviour score at age 8 years (10).

**Depressive symptoms**: measured using the short moods and feeling questionnaire (SMFQ) at age 9 years is a 13-item questionnaire designed for examining the presence of depressive symptoms in epidemiological studies and has been shown to be a strong predictor of depression. The SMFQ summarises these 13 items to give a score ranging between 0–26, where greater scores represent higher depressive symptoms (11).

**Conduct problems**: were measured using the Strengths and Difficulties Questionnaire (SDQ) at age 9 years. It is a validated and reliable assessment of dimensional child psychopathology (12).

**Maternal smoking:** was measured using a binary measure at age 8 years.

**Harmful maternal alcohol use:** at age 8 years (binary) maternal drinking includes mother’s whose weekly alcohol consumption ≥ 14 units of alcohol consumed when child is age 8 years.

**Maternal cannabis** **use**: was measured using a binary measure at age 9 years (binary).

**Maternal body mass index (BMI):** was measured at 12 weeks’ gestation.

**Birthweight:** Information was collected on birthweight and gestational age, which was used with height, weight, ethnicity of the mother and parity of the child to determine the birthweight centile of the child using the Gestation Network calculator children in the lowest 10% were considered to be small for gestational age (13).

**Gestational age:** See above.

**Maternal physical inactivity:** continuous measure of number of hours of activity per week.

**Maternal unhealthy diet:** a measure of maternal processed food intake was derived using principal components analysis (PCA) which generated a factor score at 32 weeks gestation (14).

## Deriving the adolescent cumulative risk exposure measure

We employed an area under the curve approach to summarise the amount of risk exposure reported during adolescence. This is illustrated in the figure below for a chosen participant who reported 1 cancer risk exposure at t_1_ (11.75yr), 2 exposure at t_2_ (14yr), 3 at t_3_ (16yr) and 5 at t_4_ (18.25yr). This area under the curve will enable us to rank individuals by their cumulative level of risk exposure, however will not account for the timing of the exposures themselves – for instance earlier exposure may be more detrimental for later health that later exposures.

Area under the curve =

$$\frac{Y_{1}+Y_{2}}{2}\left( t_{2}-t_{1} \right)+ \frac{Y_{2}+Y_{3}}{2}\left( t_{3}-t_{2} \right)+\frac{Y_{3}+Y_{4}}{2}\left( t_{4}-t_{3} \right)$$

Age

No. of Adolescent Cancer Risk Exposures reported

t_1_

t_2_

t_3_

t_4_

0

5

4

3

2

1

## Deriving the latent classes

A range of longitudinal mixture models were fitted to the four repeated five-category ordinal measures of adolescent cancer-risk behaviour. The least parsimonious (a longitudinal latent class analysis (LLCA)) was compared to a series of latent class growth analyses (LCGA) which incorporated the timing of the four waves (i.e. average age at response) and attempted to summarize the patterns of longitudinal change using polynomial terms.

By considering both the Bayesian Information Criterion (BIC) and face validity of competing models for a given number of classes, a cubic LCGA model was deemed most appropriate for capturing the patterns of change seen in these data. With binary data, a cubic LGCA would be mathematically equivalent to an LLCA, however with ordinal data, the former saves many parameters by making a proportional odds assumption for the relationship between class membership C and each ordinal class-indicator. LCCA is more akin to a multinomial logistic model in this regard.

Model fit statistics for a series of cubic LCGA models are shown below, both for the complete case sample of 1,951 (all four ordinal measures available) to a larger sample of 6,351 in which all participants providing one or more time points were included. These data show some degree of support for both the five- and six-class solutions, with some evidence that more classes (i.e. six) are required to adequately explain heterogeneity within the larger incomplete sample.

Table. Fit statistics for cubic LCGA models

|  |  | Complete case sample (n=1,951) | | | | Incomplete data sample (n=6,351) | | | |
| --- | --- | --- | --- | --- | --- | --- | --- | --- | --- |
| # class | # param | BIC | LMR | BLRT | Entropy | BIC | LMR | BLRT | Entropy |
| 2 | 12 | 18905.4 | N/A | N/A | 0.724 | 43659.3 | N/A | N/A | 0.625 |
| 3 | 17 | 18788.0 | < 0.0001 | < 0.0001 | 0.753 | 43344.6 | < 0.0001 | < 0.0001 | 0.546 |
| 4 | 22 | 18674.5 | < 0.0001 | < 0.0001 | 0.613 | 43180.3 | < 0.0001 | < 0.0001 | 0.523 |
| 5 | 27 | 18607.1 | 0.0001 | < 0.0001 | 0.655 | 43029.7 | < 0.0001 | < 0.0001 | 0.557 |
| 6 | 32 | 18617.7 | 0.2191 | < 0.0001 | 0.638 | 43013.1 | 0.0353 | < 0.0001 | 0.534 |
| 7 | 37 | 18635.7 | 0.3152 | 0.9920 | 0.626 | 43025.9 | 0.0570 | < 0.0001 | 0.548 |

*Bayesian Information Criterion.*  The Bayesian Information Criterion (BIC) is the traditional fit statistic for comparing mixture models. BIC will typically decrease and then increase following the incremental additional of classes. Using this statistic, the model with the lowest BIC (or other models with BIC values in that vicinity) would be deemed optimal.

*Entropy.* This is a measure of classification accuracy, and whilst it is generally of little use in determining the optimal model, it indicates the level of bias which one would expect were a standard three-step estimation to be performed.

*The Bootstrap Likelihood Ratio Test (BLRT) and the Lo-Mendell-Rubin (LMR) test statistics* both assess change in model fit when adding an additional class. Here a high p-value for a k-class model indicates no substantial improvement in fit compared to the k-1 class solution.

## Deriving the sample


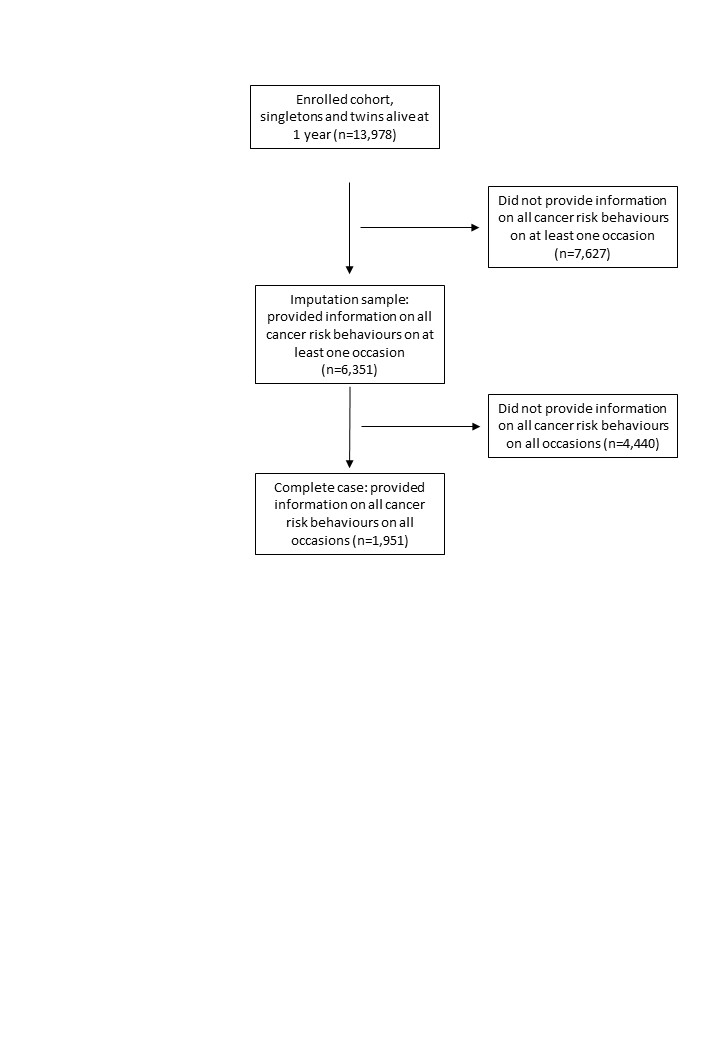


## Comparison of imputation sample and those excluded from the analysis by key demographic variables

| **Variable** | **Description** | **n** | **Excluded from analysis: Not got all data for risk behaviours at one time point (n=7,627)** | **Imputation sample: Got all data for risk behaviours at one time point (n=6,351)** | **χ 2** | **P-value** |
| --- | --- | --- | --- | --- | --- | --- |
| Sex | Female | 6,758 | 3,435 | 3,323 | 73.65 | ≤0.001 |
|  | Male | 7,200 | 4,192 | 3,028 |  |  |
| Maternal education | Degree | 1,154 | 165 | 989 | 86.53 | ≤0.001 |
|  | A-level | 1,830 | 279 | 1,551 |  |  |
|  | O-level | 2,348 | 434 | 1,914 |  |  |
|  | < O-level | 1,475 | 389 | 1,086 |  |  |
| Equivalized household income | 1^st^ quintile (highest) | 1,459 | 207 | 1,252 | 47.04 | ≤0.001 |
|  | 2^nd^ quintile | 1,355 | 218 | 1,137 |  |  |
|  | 3^rd^ quintile | 1,262 | 221 | 1,041 |  |  |
|  | 4^th^ quintile | 1,148 | 208 | 940 |  |  |
|  | 5^th^ quintile (lowest) | 975 | 240 | 735 |  |  |
| Parental social class | Professional | 1,099 | 171 | 928 | 35.30 | ≤0.001 |
|  | Managerial & technical | 2,914 | 484 | 2,430 |  |  |
|  | Skilled non-manual | 1,560 | 290 | 1,270 |  |  |
|  | Skilled manual | 916 | 225 | 691 |  |  |
| Housing tenure | Mortgaged/owned | 9,559 | 4,386 | 5,173 | 688.43 | ≤0.001 |
|  | Private rental | 2,082 | 1,563 | 519 |  |  |
|  | Subsidised rental | 1.386 | 909 | 477 |  |  |
|  |  |  |  |  |  |  |

As is shown in the above table, the subsample of ALSPAC participants selected for imputation is not a random sample: they are more likely to be female, less likely to be from the lowest income quintile; to be living in privately or subsidised rental property; to have a mother with fewer educational qualifications and have lower parental social class (p<0.001). However, for this type of bias to be present and problematic in our multivariable models would require the dependent variable (outcomes at age 24 years) to be conditionally related to whether participants are included or excluded from this analysis. The positive associations observed between these variables and selection into our sample might lead us to anticipate collider bias in the form of attenuated estimates for our exposure and outcomes. However, by conditioning on these factors (sex, income, maternal education, parental social class, housing tenure etc.) in the regression models, we propose that any residual association between our outcome and selection should be minimal and so should any (attenuating) bias.

## Risk profiles for adolescence, percentage of sample and cumulative risk score for complete case and imputed samples

| **Latent class**  **6-class solution** | **Risk profile** | **Complete case sample n=1,951** | | **Imputed Sample n=6,351** | | **Quartile** |
| --- | --- | --- | --- | --- | --- | --- |
|  |  | % of sample | Cumulative  score | % of sample | Cumulative  score |  |
| Persistent  high | Consistently high tobacco use, obesity and physical inactivity across adolescence, high then slightly decreasing alcohol use and the highest and rapidly increasing sexual risk. | 10.5 | 17.6 | 9.9 | 18.9 | 4 |
| Moderate  increasing | Consistent increase in all exposures across adolescence, with slight drop off for alcohol use, obesity and physical inactivity at age 18 years. | 7.5 | 13.1 | 8.6 | 14.2 | 4 |
| Persistent  moderate | Consistently low tobacco and sexual risk across adolescence, consistently high physical inactivity, high but rapidly declining alcohol use and consistent moderate risk of obesity. | 22.3 | 12.0 | 23.3 | 12.0 | 3 or 4 |
| Low  increasing | Consistently low tobacco and obesity, rapid increase in alcohol use and physical inactivity at age 16 and 18 and moderate sexual risk from age 14. | 13.6 | 8.5 | 11.3 | 8.4 | 2 or 3 |
| Persistent  low | Consistently low risk of all exposures across adolescence except physical inactivity. | 41 | 7.0 | 41 | 6.9 | 1 or 2 |
| Persistent  very low | Consistently very low risk of all exposures across adolescence. | 5.2 | 2.6 | 5.9 | 2.2 | 1 |

| **Latent class**  **4-class solution** | **Risk profile** | **Complete case sample n=1,951** | | **Imputed Sample n=6,351** | | **Quartile** |
| --- | --- | --- | --- | --- | --- | --- |
|  |  | % of sample | Cumulative  score | % of sample | Cumulative  score |  |
| Persistent  high | Consistently high tobacco use, obesity and physical inactivity across adolescence, high then slightly decreasing alcohol use and the highest and rapidly increasing sexual risk. | 15.4 | 16.70 | 15.0 | 17.8 | 4 |
| Persistent  moderate | Consistently low tobacco and sexual risk across adolescence, consistently high physical inactivity, high but rapidly declining alcohol use and consistent moderate risk of obesity. | 25.5 | 11.8 | 31.4 | 11.11 | 3 or 4 |
| Low  increasing | Consistently low tobacco and obesity, rapid increase in alcohol use and physical inactivity at age 16 and 18 and moderate sexual risk from age 14, increasing by age 18. | 19.9 | 9.0 | 12.8 | 9.51 | 2 or 3 |
| Persistent  low | Consistently low risk of all exposures across adolescence except physical inactivity. | 39.4 | 6.03 | 40.8 | 5.84 | 1 or 2 |

## Latent class trajectories 6-class solution

These figures show the 6-class solution for the latent classes for the complete case and (imputed sample). The purple line refers to the persistent high class who represent 10.5% (9.9%) of the sample. The blue line is the moderate increasing class and represents 7.5% (8.6%). The green line refers to the persistent moderate class who represent 22.3% (23.3%) of the sample. The yellow, orange and red lines refer to the low increasing, persistent low and persistent very low classes and represent 13.6% (11.3%), 41% (41%) and 5.2% (5.9%) of the sample, respectively.

|  |  |
| --- | --- |

## 4-class solution

These figures show the 4-class solution for the latent classes for the complete case and (imputed sample). The purple line refers to the persistent high class who represent 15.4% (15.0%) of the sample. The green line refers to the persistent moderate class who represent 25.5% (31.4%) of the sample. The yellow line refers to the low increasing class representing 19.9 (12.8%) of the sample and the red line refers to the persistently low class 39.4% (40.8) of the sample.

| 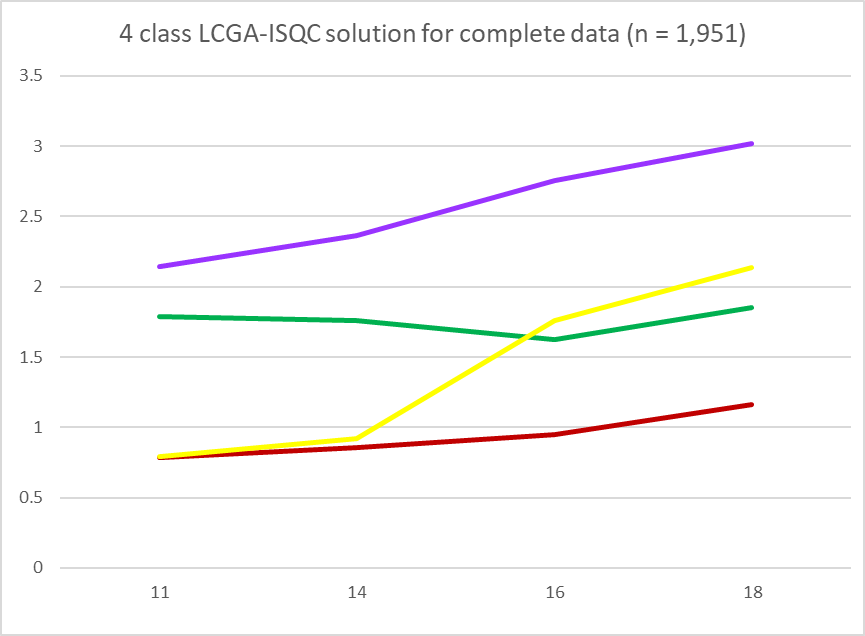 | 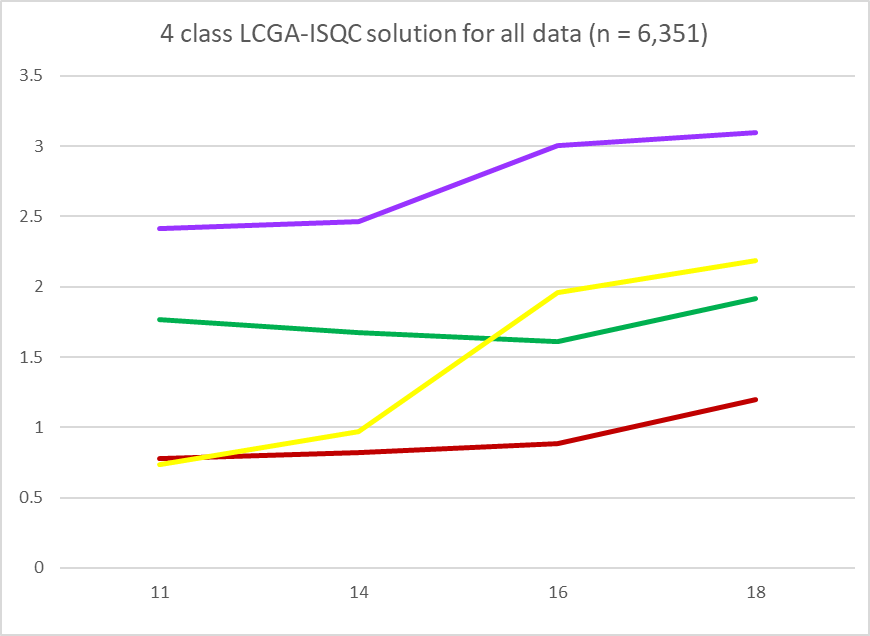 |
| --- | --- |

## Cross tabulation of cumulative risk score quartiles and proportion belonging to each of the latent classes

The tables below show cross tabulations of the two adolescent exposure measures: latent class membership and quartiles (for both the 4- and 6-class solutions). It shows that quartiles of cumulative exposure were highly consistent with the latent classes. For example, the first table shows that 100% of those in the persistent very low class belonged to the lowest quartile of the cumulative exposure measure and the second table shows that 20% of those in the lowest quartile belonged to the persistent very low class and 80% to the persistent low class.

|  |  | Cumulative risk score quartiles | | | | Sample n |
| --- | --- | --- | --- | --- | --- | --- |
|  | 6-class solution | 1 | 2 | 3 | 4 |  |
| Latent classes | Persistent very low | 1.00 |  |  |  | 122.90 |
|  | Persistent low | 0.59 | 0.41 |  |  | 810.60 |
|  | Low increasing |  | 0.35 | 0.66 |  | 265.20 |
|  | Persistent moderate |  |  | 0.70 | 0.31 | 404.70 |
|  | Moderate increasing |  |  |  | 1.00 | 93.50 |
|  | Persistent high |  |  |  | 1.00 | 254.10 |

|  |  | Cumulative risk score quartiles | | | | Sample n |
| --- | --- | --- | --- | --- | --- | --- |
|  | 6-class solution | 1 | 2 | 3 | 4 |  |
| Latent classes | Persistent very low | 0.20 |  |  |  | 122.90 |
|  | Persistent low | 0.80 | 0.78 |  |  | 810.60 |
|  | Low increasing |  | 0.22 | 0.38 |  | 265.20 |
|  | Persistent moderate |  |  | 0.62 | 0.26 | 404.70 |
|  | Moderate increasing |  |  |  | 0.20 | 93.50 |
|  | Persistent high |  |  |  | 0.54 | 254.10 |

|  |  | Cumulative risk score quartiles | | | | Sample n |
| --- | --- | --- | --- | --- | --- | --- |
|  | 4-class solution | 1 | 2 | 3 | 4 |  |
| Latent classes | Persistent low | 0.702 | 0.298 |  |  | 855.68 |
|  | Low increasing |  | 0.516 | 0.484 |  | 328.09 |
|  | Persistent moderate |  |  | 0.732 | 0.268 | 404.65 |
|  | Persistent high |  |  |  | 1.00 | 362.59 |

|  |  | Cumulative risk score quartiles | | | | Sample n |
| --- | --- | --- | --- | --- | --- | --- |
|  | 4-class solution | 1 | 2 | 3 | 4 |  |
| Latent classes | Persistent low | 1.00 | 0.60 |  |  | 855.68 |
|  | Low increasing |  | 0.40 | 0.35 |  | 328.09 |
|  | Persistent moderate |  |  | 0.65 | 0.23 | 404.65 |
|  | Persistent high |  |  |  | 0.77 | 362.59 |

## Associations between the adolescent cancer risk measure and socioeconomic status (SES) measures

|  | Cumulative adolescent risk score | |
| --- | --- | --- |
|  | Complete case¹ (n=1,951) | Imputation sample (n=6,351) |
| Socio-economic status | β [95% CI] | β [95% CI] |
| Income (n=1,826) |  |  |
| High | 9.31 [8.93,9.68] | 9.43 [9.21,9.65] |
| Middle high | 0.16 [-0.37,0.70] | 0.19 [-0.13,0.51] |
| Middle | 0.20 [-0.36,0.76] | 0.23 [-0.10,0.56] |
| Middle low | 0.95 [0.35,1.55] | 0.67 [0.33,1.02] |
| Low | 0.42 [-0.30,1.13] | 0.41 [0.04,0.77] |
| LR test p-value | p=0.03 |  |
| Linear association | 0.19 (0.04, 0.33) p=0.01 | 0.14 (0.06,0.22) p=0.001 |
| Maternal education (n=1,923) |  |  |
| Degree | 9.14 [8.76,9.53] | 9.17 [8.92,9.43] |
| A level | 0.26 [-0.26,0.77] | 0.36 [0.03,0.68] |
| O level | 0.58 [0.08,1.09] | 0.69 [0.38,1.01] |
| <O level | 1.26 [0.63,1.89] | 0.97 [0.62,1.32] |
| LR test p-value | p≤0.001 |  |
| Linear association | 0.38 (0.20,0.57) p≤0.001 | 0.32 (0.22,0.42) p≤0.001 |
| Parental social class (n=1,873) |  |  |
| Professional | 9.04 [8.63,9.45] | 9.07 [8.81,9.33] |
| Managerial and technical | 0.57 [0.08,1.06] | 0.64 [0.33,0.94] |
| Skilled non-manual | 0.59 [0.02,1.16] | 0.72 [0.38,1.06] |
| Skilled manual, etc. | 1.44 [0.69,2.19] | - 1. [0.85,1.64] |
| LR test p-value | p=0.002 |  |
| Linear association | 0.38 (0.17,0.60) p=0.001 | 0.35 (0.23,0.47) p≤0.001 |

1. The complete case analysis has a p-value for the likelihood ratio (LR) test and a p-value for the linear association generated when regressing the outcome on the exposure, using a continuous rather than a categorical exposure measure as it is shown in the table. The imputed sample has a p-value only for the linear association.

References

1. WCRF. Diet, Nutrition Physical activity and Cancer: a global Perspective Continuous Update Project Expert Report 2018. London: World Cancer Research Fund (WCRF); 2018.

2. IARC. World Cancer Report. P. K, editor. Lyon: IARC Press; 2003.

3. Wright C, Heron J, Campbell R, Hickman M, Kipping RR. Adolescent multiple risk behaviours cluster by number of risks rather than distinct risk profiles in the ALSPAC cohort. BMC Public Health. 2020;

4. Wright C, Kipping RR, Hickman M, Campbell R, Heron J. The effect of multiple risk behaviours in adolescence on educational attainment at age 16: a UK birth cohort study. BMJ Open. 2018;

5. Kipping Smith, M., Heron, J., Hickman, M., Campbell, R. RR. Multiple risk behaviour in adolescence and socio-economic status: findings from a UK birth cohort. Eur J Public Heal [Internet]. 2015;25(1):44–9. Available from: http://www.ncbi.nlm.nih.gov/pubmed/24963150

6. Campbell R, Wright C, Hickman M, Kipping RR, Smith M, Pouliou T, et al. Multiple risk behaviour in adolescence is associated with substantial adverse health and social outcomes in early adulthood: Findings from a prospective birth cohort study. Prev Med (Baltim) [Internet]. 2020;138:106157. Available from: http://www.sciencedirect.com/science/article/pii/S009174352030181X

7. Houtepen LC, Heron J, Suderman MJ, Tilling K, Howe LD. Adverse childhood experiences in the children of the Avon Longitudinal Study of Parents and Children (ALSPAC). Wellcome open Res [Internet]. 2018;3:106. Available from: https://pubmed.ncbi.nlm.nih.gov/30569020

8. Russell AE, Heron J, Gunnell D, Ford T, Hemani G, Joinson C, et al. Pathways between early-life adversity and adolescent self-harm: the mediating role of inflammation in the Avon Longitudinal Study of Parents and Children. J Child Psychol Psychiatry [Internet]. 2019/09/04. 2019;60(10):1094–103. Available from: https://pubmed.ncbi.nlm.nih.gov/31486089

9. Wechsler D, Golombok S, Rust J. WISC-IIIUK Wechsler Intelligence Scale for Children – Third Edition UK Manual. Sidcup, UK: The Psychological Corporation; 1992.

10. Loeber R, Stouthamer-Loeber M, Van Kammen WB, Farrington DP. Development of a New Measure of Self-Reported Antisocial Behavior for Young Children: Prevalence and Reliability. In: Klein MW, editor. Cross-National Research in Self-Reported Crime and Delinquency [Internet]. Dordrecht: Springer Netherlands; 1989. p. 203–25. Available from: https://doi.org/10.1007/978-94-009-1001-0_10

11. Angold A, Costello EJ, Messer SC, Pickles A. Development of a short questionnaire for use in epidemiological studies of depression in children and adolescents. Int J Methods Psychiatr Res. 1995;5(4):237–49.

12. Goodman R. Psychometric Properties of the Strengths and Difficulties Questionnaire. J Am Acad Child Adolesc Psychiatry [Internet]. 2001;40(11):1337–45. Available from: http://www.sciencedirect.com/science/article/pii/S0890856709605438

13. Flach C, Leese M, Heron J, Evans J, Feder G, Sharp D, et al. Antenatal domestic violence, maternal mental health and subsequent child behaviour: a cohort study. BJOG An Int J Obstet Gynaecol [Internet]. 2011;118(11):1383–91. Available from: https://doi.org/10.1111/j.1471-0528.2011.03040.x

14. Northstone K, Emmett P, Rogers I. Dietary patterns in pregnancy and associations with socio-demographic and lifestyle factors. Eur J Clin Nutr [Internet]. 2007/03/21. 2008;62(4):471–9. Available from: https://pubmed.ncbi.nlm.nih.gov/17375108
