# Supplementary material for: Young adult cancer risk behaviours originate in adolescence: a longitudinal analysis using ALSPAC, a UK birth cohort study
Source: BMC Cancer. 2021 Apr 7;21:365. doi: 10.1186/s12885-021-08098-8 (PMC8028717; doi:10.1186/s12885-021-08098-8)
Supplement: Supplementary file 3 — Additional file 3. Data sources. Description of data: contains both a table showing the sources of ALSPAC questionnaire and clinic data used in research and links to each questionnaire or data source. [file 12885_2021_8098_MOESM3_ESM.docx]

Table showing sources of ALSPAC questionnaire and clinic data used in research

|  | **Time 1** | **Time 2** | **Time 3** | **Time 4** |
| --- | --- | --- | --- | --- |
| **Tobacco smoking** | F10: 128 months/10.6 years  TF1: 153 months/12.75 years | CCR: 169 months/14 years  TF2: 165 months/13.75 years | CCS: 198 months/16.5 years  TF3: 185 months/15.4 years | CCT: 18+ years  TF4: 214 months/17.8 years |
| **Alcohol consumption** | TF1: 153 months/12.75 years  TF2: 165 months/13.75 years | TF2: 165 months/13.75 years | CCS: 198 months/16.5 years  TF3: 185 months/15.4 years | CCT: 18+ years  TF4: 214 months/17.8 years |
| **Obesity** | F10: 128 months/10.6 years | TF2: 165 months/13.75 years | TF3: 185 months/15.4 years | TF4: 214 months/17.8 years |
| **Sexual risk** | TF1: 153 months/12.75 years | TF2: 165 months/13.75 years | TF3: 185 months/15.4 years | TF4: 214 months/17.8 years |
| **Physical inactivity** | PUB3: 128 months/10.6 years  PUB4: 140 months/11.6 years | CCQ: 167 months/13.9 years  PUB5:157 months/13.1 years | CCS: 198 months/16.5 years  PUB7: 184 months/15.3 years | CCT: 18+ years  PUB9: 204 months/17 years |

Each of the questionnaires/clinic data used in this research are available via the following link:

<http://www.bristol.ac.uk/alspac/external/documents/ALSPAC_data_dictionary.zip>

Once you have downloaded the data dictionary each of the clinics (F10: 128 months/10.6 years; TF1: 153 months/12.75 years; TF2: 165 months/13.75 years; TF3: 185 months/15.4 years; TF4: 214 months/17.8 years & Focus 24+) can be accessed from: ALSPAC_data_dictionary.zip\built_pdf\Clinic\Child

Summary data from each of the clinics - F10: 128 months/10.6 years; TF1: 153 months/12.75 years; TF2: 165 months/13.75 years; TF3: 185 months/15.4 years; TF4: 214 months/17.8 years & Focus 24+ can be found via the following link: <http://www.bristol.ac.uk/media-library/sites/alspac/documents/researchers/clinics/focusclinicsessions.pdf>

Additionally, please find links to each of the questionnaires used:

PUB3: 128 months/10.6 years: <http://www.bristol.ac.uk/media-library/sites/alspac/migrated/documents/ques-cb22a-puberty-girl-iii.pdf> & <http://www.bristol.ac.uk/media-library/sites/alspac/migrated/documents/ques-cb22b-puberty-boy-iii.pdf>

PUB4: 140 months/11.6 years: <http://www.bristol.ac.uk/media-library/sites/alspac/migrated/documents/ques-cb24a-puberty-girl-iv.pdf> & <http://www.bristol.ac.uk/media-library/sites/alspac/migrated/documents/ques-cb24b-puberty-boy-iv.pdf>

CCR: 169 months/14 years: <http://www.bristol.ac.uk/media-library/sites/alspac/migrated/documents/ques-c20-girls-experiences-thoughts-and-behaviour.pdf>

CCQ: 167 months/13.9 years: <http://www.bristol.ac.uk/media-library/sites/alspac/migrated/documents/ques-c20-girls-experiences-thoughts-and-behaviour.pdf>

PUB5:157 months/13.1 years: <http://www.bristol.ac.uk/media-library/sites/alspac/migrated/documents/pub5-girl-quest.pdf> & <http://www.bristol.ac.uk/media-library/sites/alspac/migrated/documents/pub5-boy-quest.pdf>

CCS: 198 months/16.5 years: <http://www.bristol.ac.uk/media-library/sites/alspac/documents/questionnaires/CCS-life-of-a-16-plus-teenager.pdf>

PUB7: 184 months/15.3 years: <http://www.bristol.ac.uk/media-library/sites/alspac/migrated/documents/pub7-tf3-female-quest.pdf> & <http://www.bristol.ac.uk/media-library/sites/alspac/migrated/documents/pub7-tf3-male-quest.pdf>

CCT: 18+ years: <http://www.bristol.ac.uk/media-library/sites/alspac/migrated/documents/ques-1423-cct.pdf>

PUB9: 204 months/17 years: <http://www.bristol.ac.uk/media-library/sites/alspac/migrated/documents/pub9-female-quest.pdf> & <http://www.bristol.ac.uk/media-library/sites/alspac/migrated/documents/pub9-male-quest.pdf>
